# Effects of Perilla Seed Oil on Blood Lipids, Oxidative Stress, and Inflammation in Hyperlipidemic Rats

**DOI:** 10.3390/foods14081380

**Published:** 2025-04-17

**Authors:** Suwajee Pothinam, Chaochetdhapada Putpim, Thanyaporn Siriwoharn, Wachira Jirarattanarangsri

**Affiliations:** 1 Division of Food Science and Technology, Faculty of Agro-Industry, Chiang Mai University, Chiang Mai 50100, Thailand; suwajeepo@gmail.com; 2 Laboratory Animal Center, Office of Research Administration, Chiang Mai University, Chiang Mai 50100, Thailand; chaochetdhapada.put@cmu.ac.th

**Keywords:** inflammation, metabolic syndrome, omega-3 fatty acids, perilla seed oil

## Abstract

A high-fat diet is a key factor contributing to hyperlipidemia. Perilla seed oil, a plant-based source of omega-3, has the potential to reduce this risk. However, its effects have not been fully established. This study aimed to evaluate the effects of perilla seed oil on blood lipid levels, oxidative stress, and inflammation in rats induced with hyperlipidemia through a high-fat diet. Male Wistar rats were administered perilla seed oil at a dosage of 0.67 g/kg body weight per day for 8 weeks. The results showed that perilla seed oil significantly reduced triglyceride levels by 38.00% and 41.88% and total cholesterol levels by 17.16% and 15.91% in the high-fat diet and normal diet groups, respectively (*p* < 0.05). However, perilla seed oil had no significant effect on HDL and LDL levels. Additionally, perilla seed oil supplementation significantly reduced malondialdehyde (MDA) levels, a biomarker of oxidative stress, by 68.18% in the high-fat diet group and 29.72% in the normal diet group. Regarding its anti-inflammatory effects, perilla seed oil reduced interleukin-6 (IL-6) levels by 15.21% and 64.27% in the high-fat diet and normal diet groups, respectively (*p* < 0.05). These findings suggest that perilla seed oil has the potential to reduce the risk of metabolic syndrome.

## 1. Introduction

The prevalence of metabolic syndrome has been increasing globally [1]. Metabolic syndrome is a cluster of metabolic abnormalities commonly associated with dyslipidemia, high blood pressure, and abdominal obesity [2]. This condition not only increases the risk of cardiovascular diseases, but is also linked to elevated oxidative stress and inflammation [3]. Obesity and dyslipidemia are key contributors to metabolic syndrome, leading to complications such as hyperlipidemia and atherosclerosis, which further elevate the risk of cardiovascular diseases, hypertension, and type 2 diabetes [4].

Unhealthy dietary habits are one of the primary factors contributing to metabolic syndrome. Fat is a major macronutrient, and the type and proportion of fatty acids consumed significantly impact overall health and metabolic function [5]. The n-6/n-3 polyunsaturated fatty acid (PUFA) ratio plays a crucial role in the development of metabolic disorders [6]. Consuming a high omega-6 to omega-3 ratio, which is common in the modern Western diet, tends to promote a higher level of inflammation and contributes to chronic low-grade inflammation [7]. This condition is associated with the development of various diseases, including cardiovascular diseases, cancer and inflammatory diseases [8]. Omega-3 intake has the potential to reduce these risks, particularly for individuals with a high omega-6 intake, as well as those with dyslipidemia or metabolic syndrome-related risks. Evidence suggests that regular consumption of omega-3 fatty acids can reduce the risk of cardiovascular disease-related mortality [9,10]. Omega-3 fatty acids primarily consist of eicosapentaenoic acid (EPA) and docosahexaenoic acid (DHA), which exhibit anti-inflammatory properties, inhibit platelet aggregation, regulate blood lipid profile and cholesterol levels, and lower blood pressure [11]. A study by Yashodhara et al. [12] reported that individuals who regularly consume omega-3-rich fish have a 17% lower risk of cardiovascular disease-related mortality. Tousoulis et al. [13] found that omega-3 fatty acids can reduce total cholesterol, LDL, and triglyceride levels while improving endothelial function and reducing arterial stiffness in individuals with metabolic syndrome. Lima Rocha et al. [14] reported that fish oil supplementation can reduce triglycerides in the liver and plasma, as well as liver tissue damage in rats fed a high-fat diet.

Perilla seed oil is a rich source of polyunsaturated fatty acids, particularly omega-3 fatty acids, in the form of alpha-linolenic acid (ALA). It also contains omega-6 and omega-9 fatty acids. Our previous study [15] found that the most abundant fatty acid in perilla seed oil is alpha-linolenic acid (56.94–58.02%), followed by linoleic acid (18.10–18.37%), oleic acid (12.86–13.38%), palmitic acid (7.06–7.52%), and stearic acid (3.28–3.55%). ALA, a plant-based omega-3 fatty acid, serves as an alternative source for individuals allergic to fish-derived products or those following a vegetarian diet. ALA can be converted into EPA and DHA, and multiple studies have shown that ALA exhibits lipid-lowering effects similar to those of EPA and DHA [16]. Among vegetable oils, perilla seed oil contains the highest ALA content (54–64%) [17]. Due to its high omega-3 content, perilla seed oil has been shown to reduce cholesterol and triglyceride levels, thereby lowering the risk of ischemic heart disease and atherosclerosis. Additionally, perilla seed oil contains phenolic compounds such as rosmarinic acid, apigenin, and luteolin [18], which possess anti-inflammatory and antioxidant properties, further reducing the risk of atherosclerosis.

Although perilla seed oil has significant health benefits, its consumption remains limited, possibly due to the lack of extensive scientific research supporting its therapeutic or preventive effects on various diseases, as well as insufficient safety information. Therefore, this study aims to investigate the effects of perilla seed oil on blood lipid levels, oxidative stress (MDA), and inflammation (IL-6) using an animal model. The findings from this study will serve as preliminary data for evaluating the potential of perilla seed oil as a functional food for human consumption in the future.

## 2. Materials and Methods

### 2.1. Preparation of Perilla Seed Oil

Perilla seeds were collected from Mae Fah Luang district, Chiang Rai, Thailand, in December 2022. The oil was extracted using supercritical carbon dioxide extraction at a temperature of 60 °C and a pressure of 220 bar, following the method described in our previous study [15]. The fatty acid composition of perilla seed oil was measured according to the method of Morrison and Smith [19] using gas chromatography (Bruker, Scion 436-GC, Germany) equipped with an RT^®^-2560 column (biscyanopropyl polysiloxane) (100 m × 0.25 mm ID, 0.2 µm df) (Restek^®^, Bellefonte, PA, USA) and a flame ionization detector (FID). The most abundant fatty acid in perilla seed oil was α-linolenic acid (57.38 ± 0.10%), followed by linoleic acid (18.37 ± 0.12%), oleic acid (13.17 ± 0.04%), palmitic acid (7.14 ± 0.10%), and stearic acid (3.46 ± 0.07%).

### 2.2. Animals

Twenty-four 8-week-old male Wistar rats (180–200 g) were obtained from Nomura Siam International Co., Ltd., Bangkok, Thailand. The sample size was calculated using G*Power software (version 3.1.9.6, Heinrich-Heine-Universität Düsseldorf, Düsseldorf, Germany) based on data from Thomas et al. [20]. The animal experiments and all procedures were approved by the Ethics Committee of the Laboratory Animal Center, Chiang Mai University, Thailand, under approval number 2566/RT0019, dated 25 September 2023.

### 2.3. Experimental Design

The rats were housed in individual cages under controlled conditions (21 ± 1 °C, 50 ± 10% humidity, and a 12/12 h dark/light cycle). They were provided ad libitum access to food and water for one week to acclimatize. Before the experiment began, the rats were fasted for 12 h to collect blood samples for measuring blood lipid levels, malondialdehyde (MDA), and interleukin-6 (IL-6). The blood was centrifuged at 2000 rpm at 4 °C for 10 min, and the serum was separated and frozen at −80 °C until analysis. After blood collection, each rat was subcutaneously administered 2 mL of 0.9% normal saline solution as a fluid replacement.

Weeks 1–12: The rats were divided into two groups.

Group 1 (ND, *n* = 12): a normal diet (Smart Heart Hamster Food, Complete and Balanced formula; 54.5% carbohydrates, 24.0% protein, 4.5% fat, 5.0% fiber, and 12.0% other components).

Group 2 (HFD, *n* = 12): a high-fat diet (HFD) formulated with lard based on Pratchayasakul et al. (2011), consisting of 57.60% fat, 26.45% protein, 14.27% carbohydrates, and 1.68% cholesterol [21].

The diets were provided for 12 weeks. Body weight and food consumption were recorded weekly. At the end of the 12th week, the rats were fasted for 12 h, and blood samples were collected to measure blood lipid levels, MDA, and IL-6. After blood collection, the rats received 2 mL of 0.9% normal saline solution subcutaneously.

Weeks 13–20: After the 12-week induction period to establish hyperlipidemia, the rats were further divided into different treatment groups (*n* = 6). Rats in both the ND and HFD groups were administered either lard or perilla seed oil at a dose of 0.67 g per kg of body weight for 8 weeks as follows:

Group 1: ND + lard (0.67 g/kg BW).

Group 2: ND + perilla seed oil (0.67 g/kg BW).

Group 3: HFD + lard (0.67 g/kg BW).

Group 4: HFD + perilla seed oil (0.67 g/kg BW).

Body weight, food, and water consumption were recorded weekly. At the end of the 20th week, the rats were fasted for 12 h and euthanized using thiopental sodium (200 mg/kg BW). Blood samples were collected from the abdominal vein to measure blood lipid levels, MDA, and IL-6. The blood was processed as described previously, and the serum was frozen at −80 °C until analysis.

### 2.4. Blood Lipid Measurement

Blood lipid levels, including total cholesterol, HDL, and triglycerides, were analyzed using a Dri-Chem NX 500 automated clinical chemistry analyzer (Fujifilm, Tokyo, Japan), following the manufacturer’s protocol. LDL analysis was performed at the Center for Veterinary Clinical Pathology and Animal Health Innovation, Faculty of Veterinary Medicine, Chiang Mai University, using an automated chemistry analyzer, model BX-3010 (Sysmex, Kobe, Japan), according to the laboratory’s standard procedures.

### 2.5. Malondialdehyde (MDA) Measurement Using the TBARs Method

Blood MDA levels were analyzed using a modified method based on Ohkawa et al. [22]. A 100 µL serum sample was mixed with 1.5 mL of 0.8% thiobarbituric acid (TBA), followed by the addition of 0.2 mL of 8.1% sodium dodecyl sulfate (SDS) and 1.5 mL of 20% acetic acid. The mixture was thoroughly mixed and adjusted to a final volume of 5 mL with distilled water. The sample was then incubated in a water bath at 95 °C for 1 h. After cooling, 5 mL of a butanol-pyridine mixture (15:1 ratio) was added, and the sample was centrifuged at 4000 rpm for 10 min. The absorbance of the upper clear phase was measured at 532 nm and compared to a standard curve of 1,1,3,3-Tetraethoxypropane (TEP). The results were reported as micromoles per liter.

### 2.6. Interleukin-6 (IL-6) Measurement

IL-6 levels were determined using the Rat IL-6 Uncoated ELISA Kit (Thermo Fisher Scientific, Waltham, MA, USA), following the manufacturer’s protocol. Briefly, the ELISA plate was coated with 100 µL of capture antibody and incubated at 4 °C for 16 h, followed by the addition of 250 µL of blocking buffer for another 16 h. IL-6 standards (31.25–2000 pg/mL) were prepared using 2-fold serial dilution, and 50 µL of samples were added to the plate. After a 2 h incubation at room temperature, 50 µL of detection antibody was added, followed by 100 µL of streptavidin-HRP. The plate was then incubated for 1 h, and 100 µL of substrate solution was added for 15 min. The reaction was stopped by adding 100 µL of 2N H_2_SO_4_. The absorbance was measured at 450 nm, and IL-6 concentrations were determined using the standard curve and expressed in pg/mL.

### 2.7. Statistical Analysis

Data are expressed as means ± standard error of the mean (SEM). The experiment was designed using a randomized complete block design (RCBD), and mean differences were compared using Duncan’s multiple range test (DMRT) at a significance level of *p* < 0.05. Statistical analysis was performed using SPSS version 17.0 (SPSS Inc., Chicago, IL, USA).

## 3. Results and Discussion

### 3.1. Effects on Body Weight

**Figure 1:**
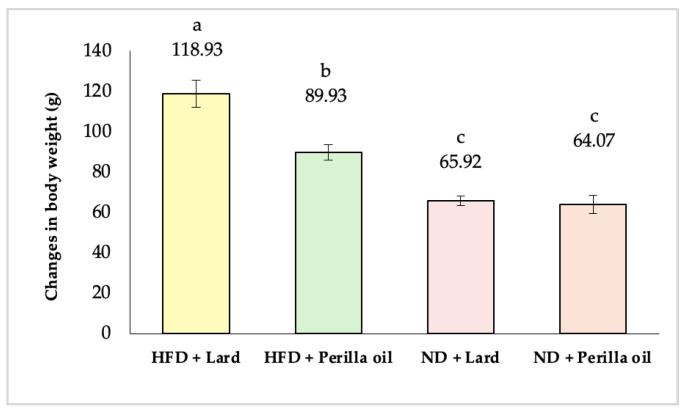
Changes in body weight of rats before and after receiving lard or perilla seed oil. HFD = high-fat diet; ND = normal diet. Different letters indicate significant differences (*p* < 0.05).

Changes in body weight are key indicators of obesity and metabolic syndrome. As shown in Figure 1, after 8 weeks of supplementation with either lard or perilla seed oil, all four groups of rats gained weight. However, the HFD group supplemented with perilla seed oil had significantly lower weight gain than the HFD group supplemented with lard (*p* < 0.05). The rats in the HFD-lard group gained 118.93 g, representing a 20.44% increase, whereas those in the HFD-perilla seed oil group gained only 89.93 g, a 17.64% increase. This is because omega-3 fatty acids could reduce adiposity by improving lipid metabolism, such as stimulating lipolysis, inhibiting lipogenesis in the liver, enhancing fatty acid oxidation in muscles, and decreasing lipid accumulation in adipocytes [23]. The results are consistent with Zhang et al. [24], who assessed the sub-chronic oral toxicity of perilla seed oil over a 90-day period in Wistar rats. Their findings showed that rats receiving 16 g/kg of perilla seed oil exhibited less weight gain and a slight decrease in food intake. These results suggest that perilla seed oil administration can inhibit weight gain in rats. Additionally, other studies have demonstrated that oils rich in alpha-linolenic acid, such as chia seed oil [25] and flaxseed oil [26], can also inhibit weight gain in experimental rats. However, there was no significant difference in weight gain between the ND groups supplemented with either lard or perilla seed oil.

### 3.2. Effects on Blood Lipid Levels

Triglycerides are nonpolar lipid molecules composed of a glycerol backbone and three fatty acid molecules. They serve as the primary form of fat storage and an energy reserve in living organisms [27]. Elevated triglyceride levels are a contributing factor that can increase the risk of cardiovascular disease [28].

**Figure 2:**
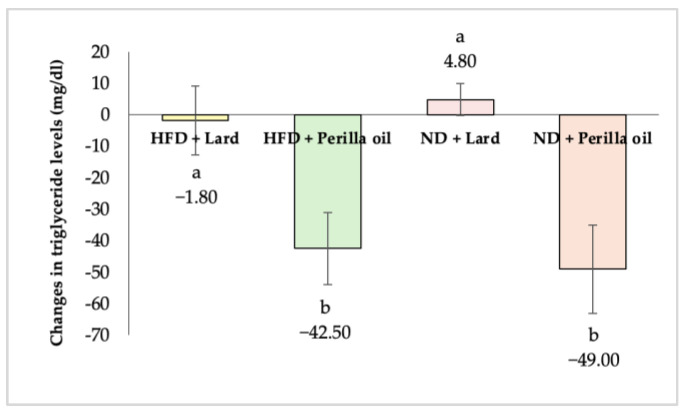
Changes in triglyceride levels in rats before and after receiving lard or perilla seed oil. HFD = high-fat diet; ND = normal diet. Different letters indicate significant differences (*p* < 0.05).

As shown in Figure 2, after 8 weeks of supplementation with either lard or perilla seed oil, rats in both the normal diet (ND) and high-fat diet (HFD) groups that received perilla seed oil exhibited a significant reduction in triglyceride levels (*p* < 0.05). The HFD-perilla seed oil group showed a triglyceride reduction of 42.50 mg/dL (38.00%), while the ND-perilla seed oil group exhibited a reduction of 49.00 mg/dL (41.88%).

The triglyceride-lowering effects of omega-3 fatty acids are primarily attributed to their role in reducing very-low-density lipoprotein (VLDL) synthesis in the liver. The underlying mechanisms include decreasing fatty acid availability for triglyceride synthesis by reducing de novo lipogenesis, enhancing fatty acid β-oxidation, reducing free fatty acid transport to the liver, decreasing hepatic enzyme activity for triglyceride synthesis (diacylglycerol acyltransferase or phosphatidic acid phosphohydrolase), and increasing hepatic phospholipid synthesis instead of triglyceride [29,30]. It has been reported that male hamsters receiving alpha-linolenic acid (ALA) instead of oleic acid exhibited a 45% reduction in blood triglyceride levels. This reduction occurs when the amount of alpha-linolenic acid in the diet reaches 10% of the total fatty acid content, with no further decrease observed at higher intake levels. The decrease in plasma triglycerides was associated with a reduction in the activity of two lipogenic enzymes, namely ME (malic enzyme) and ACC (acetyl-CoA carboxylase) [31].

Cholesterol is a crucial lipid component of cell membranes and serves as a precursor for various steroid hormones [32]. It plays an essential role in cellular function throughout the body. However, elevated blood cholesterol levels can have negative effects on health. High cholesterol is a major risk factor for the formation of arterial plaques, which increase the risk of developing conditions such as coronary artery disease, aortic aneurysm, and stroke [33].

**Figure 3:**
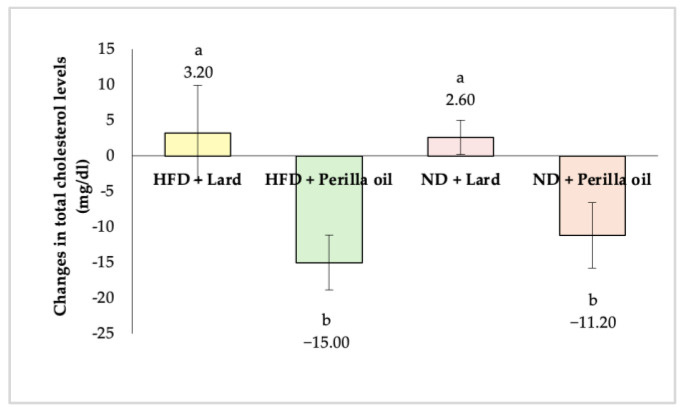
Changes in total cholesterol levels in rats before and after receiving lard or perilla seed oil. HFD = High-fat diet; ND = normal diet. Different letters indicate significant differences (*p* < 0.05).

As shown in Figure 3, after 8 weeks of supplementation with either lard or perilla seed oil, rats in both the high-fat diet (HFD) and normal diet (ND) groups that received perilla seed oil exhibited a significant reduction in total cholesterol levels (*p* < 0.05). The HFD-perilla seed oil group showed a total cholesterol reduction of 15.00 mg/dL (17.16%), while the ND-perilla seed oil group exhibited a reduction of 11.20 mg/dL (15.91%). Additionally, lard supplementation did not significantly affect total cholesterol levels in either the HFD or ND groups (*p* ≥ 0.05).

Replacing saturated fatty acids with polyunsaturated fatty acids in the diet has been shown to lower total cholesterol levels. Omega-3 fatty acids reduce the activity or expression of HMG-CoA Reductase, which reduces cholesterol production in the liver, resulting in a decrease in total cholesterol levels in the blood [34]. Perilla seed oil has been found to increase the levels of the enzymes p-AMPK (phosphorylated AMP-activated protein kinase) and p-ACC (phosphorylated acetyl-CoA carboxylase) in the livers of experimental animals, both of which are enzymes that can inhibit the synthesis of cholesterol and fatty acids [35]. Additionally, perilla seed oil was shown to reduce total cholesterol (TC), low-density lipoprotein (LDL), and triglyceride (TG) levels in 7-month-old male New Zealand white rabbits with acute hyperlipidemia induced by a high-cholesterol diet. The reduction was found to be dose-dependent and did not affect HDL levels. Furthermore, similar results were observed in the group receiving lovastatin [36].

High-density lipoprotein (HDL) is a type of lipoprotein in plasma that transports cholesterol, phospholipids, and apolipoproteins. Several mechanisms have been reported to explain how HDL can reduce the formation of vascular lesions. One key mechanism is its role in reverse cholesterol transport, in which cholesterol is transported from tissues and cells back to the liver for biliary excretion [37,38].

**Figure 4:**
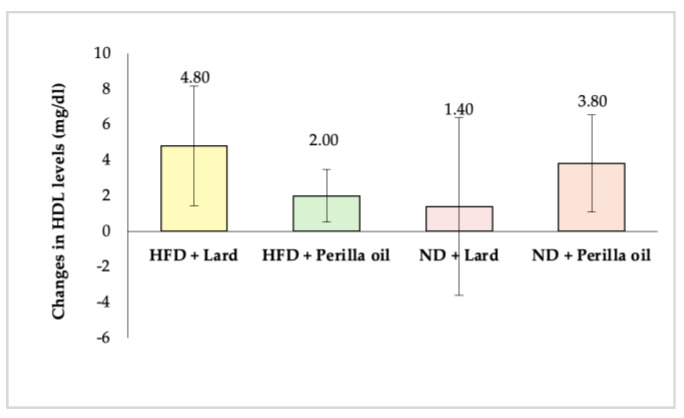
Changes in HDL levels in rats before and after receiving lard or perilla seed oil. HFD = high-fat diet; ND = normal diet.

As shown in Figure 4, after 8 weeks of supplementation with either lard or perilla seed oil, there were no significant changes in HDL levels among any of the experimental groups (*p* ≥ 0.05). This suggests that perilla seed oil did not have a significant effect on HDL levels (*p* ≥ 0.05).

Low-density lipoprotein (LDL) is the primary lipoprotein responsible for transporting cholesterol in plasma and plays a crucial role in the development of atherosclerosis. Elevated LDL levels are associated with an increased risk of cardiovascular disease [39].

**Figure 5:**
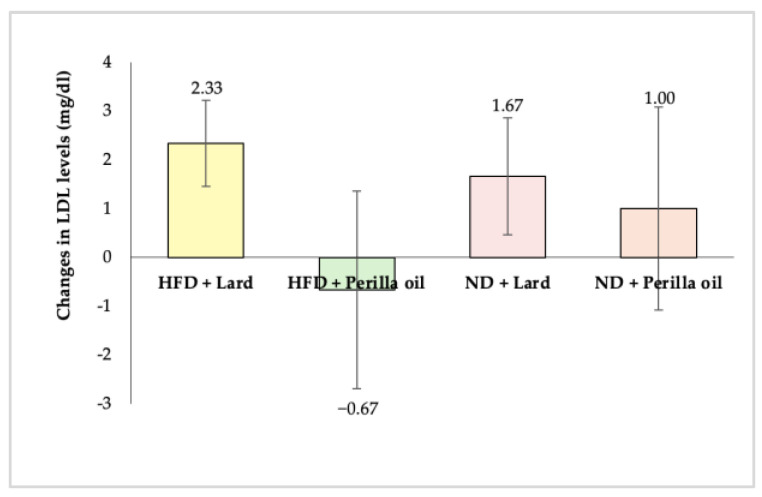
Changes in LDL levels in rats before and after receiving lard or perilla seed oil. HFD = high-fat diet; ND = normal diet.

As shown in Figure 5, after 8 weeks of supplementation with either lard or perilla seed oil, there were no significant changes in LDL levels among any of the experimental groups (*p* ≥ 0.05). This indicates that perilla seed oil did not have a significant effect on LDL levels (*p* ≥ 0.05).

Our study found that administration of perilla oil at a dose of 0.67 g/kg body weight for 8 weeks in hyperlipidemic rats had no significant effect on HDL and LDL levels. However, several studies have reported that vegetable oils that are high in ALA, at higher doses than used in this experiment, can increase HDL levels in the blood. For example, supplementation with flaxseed oil at 10 mg/kg in rats fed a high-fat diet can significantly increase HDL levels [40]. In contrast, the group of rats fed a diet containing 23.5% perilla oil for 16 weeks had significantly reduced HDL levels compared to the control group [41]. Due to the variety of experimental conditions and results, further studies are needed to investigate the factors that may affect the HDL response to omega-3 fatty acid supplementation and to better understand the mechanisms involved.

### 3.3. Antioxidant Activity Assessment

The measurement of malondialdehyde (MDA) is widely used as a biomarker for assessing oxidative stress in the pathology of various diseases. Lipid oxidation is a chain reaction phenomenon that leads to the formation of reactive compounds, causing cellular damage [42]. Free radicals are continuously generated in the human body during metabolic processes. However, excessive production of these radicals can lead to damage to biomolecules such as DNA, lipids, and proteins, which is associated with an increased risk of diseases such as cardiovascular disease and cancer [43]. Elevated MDA levels indicate increased oxidative stress.

**Figure 6:**
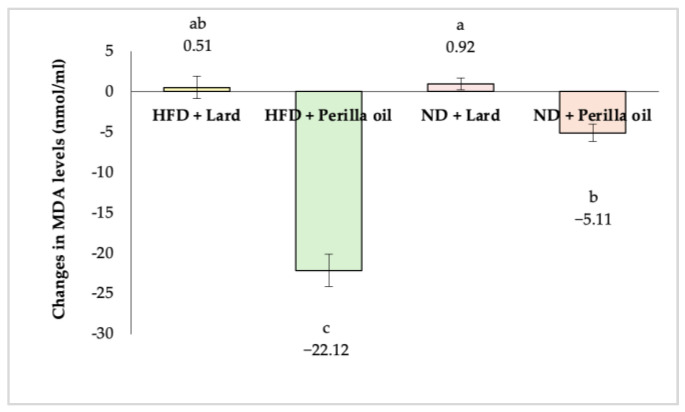
Changes in MDA levels in rats before and after receiving lard or perilla seed oil. HFD = high-fat diet; ND = normal diet. Different letters indicate significant differences (*p* < 0.05).

As shown in Figure 6, after 8 weeks of supplementation with either lard or perilla seed oil, MDA levels in the serum of rats that received perilla seed oil significantly decreased (*p* < 0.05). In the high-fat diet group, MDA levels were reduced by 22.12 nmol/mL (68.18%), while in the normal diet group, MDA levels decreased by 5.11 nmol/mL (29.72%).

The antioxidant capacity of perilla seed oil is primarily derived from ALA, which exhibits strong antioxidant and free radical scavenging properties, protecting against cellular damage, apoptosis, and inflammatory responses [44]. Additionally, perilla seed oil is rich in various phenolic compounds, including rosmarinic acid, rosmarinic acid-3-O-glucoside, caffeic acid, ferulic acid, and caffeic acid-3-O-glucoside [45]. These natural antioxidants help neutralize excess free radicals. The antioxidant activity of phenolic compounds is attributed to their redox properties, which allow them to act as reducing agents, hydrogen donors, singlet oxygen quenchers, or metal chelators [43]. Our previous study demonstrated that perilla seed oil exhibits notable antioxidant properties, as indicated by its IC_50_ values for DPPH (9.93–13.27 mg/mL) and ABTS (141.81–178.79 mg/mL). Additionally, perilla seed oil contains a high level of tocopherols (552.78–707.91 mg/kg), particularly gamma-tocopherol, contributing to its oxidative stability and antioxidant potential [15]. Han et al. [46] reported that flaxseed oil could reduce MDA concentrations in rats fed a high-fat diet, indicating its protective effect against oxidative stress. This effect may be attributed to its ability to reduce free radical production or enhance free radical scavenging activity.

### 3.4. Anti-Inflammatory Activity

To evaluate the effects of perilla seed oil on inflammation, IL-6 (Interleukin-6) levels were measured. IL-6 is a cytokine involved in immune response and inflammation [47]. It is moderately associated with certain risk factors, including smoking, diabetes, and dyslipidemia, and also correlates with several downstream inflammatory markers, highlighting its role in promoting inflammatory responses [48].

**Figure 7:**
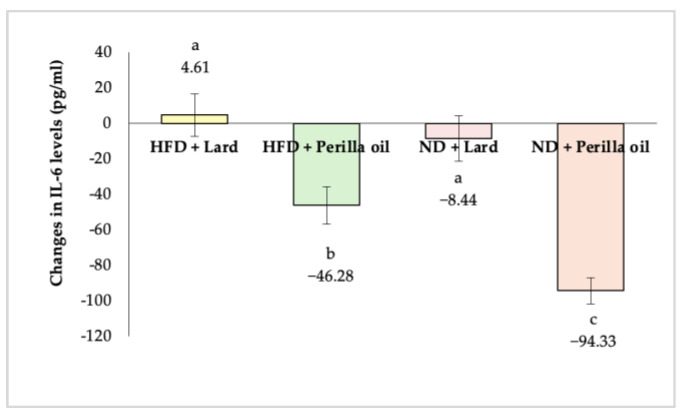
Changes in IL-6 levels in rats before and after receiving lard or perilla seed oil. HFD = high-fat diet; ND = normal diet. Different letters indicate significant differences (*p* < 0.05).

As shown in Figure 7, after 8 weeks of supplementation with either lard or perilla seed oil, significant reductions in blood IL-6 levels (*p* < 0.05) were observed in both the high-fat diet and normal diet groups that received perilla seed oil. Notably, IL-6 levels in the normal diet group decreased more significantly (by 94.33 pg/mL or 64.27%) compared to the high-fat diet group (by 46.28 pg/mL or 15.21%). These findings indicate that 0.67 g/kg/day of perilla oil effectively lowers IL-6 levels, particularly in the normal diet group.

The anti-inflammatory capacity of perilla oil comes from the anti-inflammatory effect of ALA. When ALA is consumed from food, it is metabolized through desaturation and elongation processes to form a 20-carbon fatty acid, eicosapentaenoic acid (EPA). Meanwhile, linoleic acid (n-6) is converted into arachidonic acid. Arachidonic acid is the precursor of PGH2, which is further converted into PGE2 and TXA2, compounds that play a role in promoting inflammation and blood clotting through the cyclooxygenase enzyme. EPA has the ability to inhibit the conversion of linoleic acid (n-6) into arachidonic acid through competitive inhibition [49], as arachidonic acid is the precursor of inflammatory mediators (eicosanoids), which play an important role in the inflammatory process and immune system response. Therefore, ALA has anti-inflammatory properties by reducing the production of cytokines, lipids, and lipoproteins that can cause inflammation [49]. Previous studies have shown that flaxseed oil, which is high in ALA, can reduce the levels of inflammatory cytokines IL-6, TNF-α, and MCP-1 in the plasma of apoE-KO mice fed a high-fat diet [46]. Another study showed that perilla oil can significantly improve colonic inflammation induced by a high-fat diet by reducing pro-inflammatory cytokines in serum and the colon, such as interleukin IL-1β, IL-6, and TNF-α [20].

High-fat diet (HFD) feeding in rats has been proven to be a useful model for studying the potential effects of dietary fat intake in humans. Therefore, the rat model is a useful tool for inducing obesity, as rats gain weight rapidly when fed a high-fat diet [50]. Scientific evidence indicates that consuming a diet high in saturated fat is a factor that contributes to dyslipidemia, obesity, and metabolic syndrome [51,52]. Additionally, obesity has been shown to be associated with markers of oxidative stress, including elevated levels of reactive oxygen species (ROS). The oxidative status in obesity is closely linked to the secretion of pro-inflammatory cytokines, while these pro-inflammatory cytokines can further induce oxidative stress [53]. Our study found that perilla seed oil supplementation at 0.67 g/kg body weight for 8 weeks significantly reduced total cholesterol and triglyceride concentrations in hyperlipidemic rats. These findings suggest that perilla oil is a potential source of omega-3 fatty acids and exerts beneficial effects on blood lipid levels, oxidative stress, and inflammation, which are risk factors for various chronic diseases. However, further studies are needed to determine the appropriate dosage and evaluate the efficacy of perilla oil in disease prevention. Clinical studies in humans are also necessary to validate these findings and establish the appropriate dosage for various applications, such as in medicine, the food industry, and the cosmetic industry.

## 4. Conclusions

The present study demonstrated that perilla seed oil supplementation at 0.67 g/kg body weight for 8 weeks significantly improved dyslipidemia by reducing triglyceride and total cholesterol levels. Furthermore, it exhibited potent antioxidant and anti-inflammatory effects, as evidenced by significant reductions in malondialdehyde (MDA) and interleukin-6 (IL-6) levels. These benefits are partly attributed to the presence of bioactive compounds, including ALA, tocopherols, and phenolic compounds. In summary, these findings suggest that perilla seed oil has strong potential as a functional food for reducing the risk of metabolic syndrome-related diseases.

## Data Availability

The original contributions presented in this study are included in the article. Further inquiries can be directed to the corresponding authors.

## Abbreviations

The following abbreviations are used in this manuscript:

ALA	Alpha-linolenic acid
HDL	High-density lipoprotein
LDL	Low-density lipoprotein
MDA	Malondialdehyde
